# Structural and Functional Alterations in Betel-Quid Chewers: A Systematic Review of Neuroimaging Findings

**DOI:** 10.3389/fpsyt.2019.00016

**Published:** 2019-01-29

**Authors:** Adellah Sariah, Zhening Liu, Weidan Pu, Haihong Liu, Zhimin Xue, Xiaojun Huang

**Affiliations:** ^1^Mental Health Institute of the Second Xiangya Hospital, Central South University, Changsha, China; ^2^Department of Mental Health and Psychiatric Nursing, Hubert Kairuki Memorial University, Dar es Salaam, Tanzania; ^3^Medical Psychological Institute, Second Xiangya Hospital, Central South University, Changsha, China; ^4^Mental Health Center of Xiangya Hospital, Central South University, Changsha, China

**Keywords:** betel quid, resting state, functional MRI, structural MRI, betel quid dependence, diffusion tensor imaging (DTI), systematic review, brain alterations

## Abstract

**Background:** A number of neuroimaging studies have investigated structural, metabolic, and functional connectivity changes in betel quid (BQ) chewers. We present a systematic review of neuroimaging studies with emphasis on key brain systems affected by BQ chewing to bring a better understanding on the neuro mechanisms involved in BQD.

**Methods:** All BQ neuroimaging studies were identified by searching PubMed, EMBASE, and Google scholar for English articles published until March 2018 using the key words: Betel-quid, resting state, functional MRI, structural MRI, diffusion tensor imaging (DTI), and betel quid dependence basing on the PRISMA criteria. We also sought unpublished studies, and the rest were obtained from reference lists of the retrieved articles. All neuroimaging studies investigating brain structural, and functional alterations related to BQ chewing and BQ dependence were included. Our systematic review registration number is CRD42018092669.

**Results:** A review of 12 studies showed that several systems in the brain of BQ chewers exhibited structural, metabolic, and functional alterations. BQ chewing was associated with alterations in the reward [areas in the midbrain, and prefrontal cortex (PFC)], impulsivity (anterior cingulate cortex, PFC) and cognitive (PFC, the default mode, frontotemporal, frontoparietal, occipital/temporal, occipital/parietal, temporal/limbic networks, hippocampal/hypothalamus, and the cerebellum) systems in the brain. BQ duration and severity of betel quid dependence were associated with majority of alterations in BQ chewers.

**Conclusion:** Betel quid chewing is associated with brain alterations in structure, metabolism and function in the cognitive, reward, and impulsivity circuits which are greatly influenced by duration and severity of betel quid dependence.

## Introduction

Betel quid (BQ) is a chewable substance made up of fresh unripe or dried Areca catechu nut (AN) usually wrapped in a betel leaf from the piper betel vine, smeared with aqueous lime, and always flavored (1). Worldwide, BQ ranks number four among the most popular self-administered psychoactive substances (caffeine, alcohol, and nicotine) (2). Studies have found that, more than 600 million people use BQ within the Indo-Asia-Pacific regions (3) and Asian immigrants in Africa, Europe, and North America (2). The proportion of betel quid dependence (BQD) among users was 20.9–33.3% in mainland China and Sri Lanka; 41.3–52.8% in Taiwan and Malaysia; and 84.4–99.6% in Indonesia and Nepal. Generally, men displayed a higher BQD prevalence (3.5–7.7%) than women (0.3–1.1%) in Taiwan and mainland China, while the opposite was observed in Malaysia and Indonesia, where the prevalence was higher in women (7.7–40.5%) than in men (2.0–10.0%) (4).

Habitual users of have reported experiencing psychological effects immediately after chewing BQ; including heightened alertness, euphoria, relaxation, arousal, anti-migraine, improved motor responses, and a sense of wellbeing (5). Onset of such effects was observed within 2 min after chewing, suggesting that the active compounds of BQ are immediately absorbed in the mucosal membrane of the oral cavity. Fresh and occasional BQ users tend to experience stronger effects than habitual chewers, proposing that tolerance occurs with continual use (6). For some, the initial BQ taste may be unpleasant, however the experienced cognitive effects such as arousal and increased alertness may be considered pleasant enough for a repetitive behavior that results into dependence (7). Similar to opiate withdrawal, BQ dependent users have acknowledged experiencing tolerance, craving, substance seeking behavior, and withdrawal symptoms (4, 8–12), that meet the criteria for substance abuse (13). Such symptoms originate from chronic exposure of G protein coupled receptors to compounds like arecoline, leading to reduced receptor sensitivity (14, 15) which parallels the development of tolerance and habitual drug use (7). Arecoline is one of the AN alkaloids found in betel preparations, others include guvacine, guvacoline, and arecaidine (16). Previous studies considered arecoline as the main ingredient of AN that is responsible for numerous symptoms of BQ chewing (17). However, recent studies have reported a higher concentration of arecoline and guvacine in young and mature betel nut respectively (16). Arecoline in BQ activates the M_5_ muscarinic acetylcholine receptors (mAChR) which in turn potentiate dopamine (DA) transmission in the nucleus accumbens (NAc) (18) and projects to the dorsal striatum for regulation of synaptic plasticity which influences striatal microcircuitry (19). Similar to nicotine's mechanism of action, BQ through the action of arecoline is thought to enhance excitatory input to DA neurons via presynaptic activation (20). Arecoline also affects smooth muscles and binds to GABA receptors in the brain, and thus contributing to the reported psychoactive effects (2). Meanwhile, cholinergic (nicotinic and muscarinic) and inhibitory GABA'ergic input also exert a high modulatory effect on mesolimbic dopaminergic neurons which are involved in reinforcement learning, reward processing (21), and addiction (22). Additionally, the ventral tegmental area (VTA), NAc and the prefrontal cortex (PFC) form the mesocorticolimbic system which is the primary reward pathway, known to increase DA concentration in the VTA and other projection areas directly and/or indirectly (20). Long term use of psychoactive substances is often associated with the disruption of the DA reward system (23), and adaptation to repeated drug use is often accompanied by brain changes (in structure, neurons, receptors, molecular mechanisms, and connectivity) (24) and by memories formed from the experienced drug intoxication (25). For example, people addicted to substances experience a reduced sensitivity to brain's reward system due to decreased D2 receptors compared to non-addicts (26). The loss in reward sensitivity may explain the reported compulsion to continue taking drugs in order to regain the pleasurable feelings once experienced from the reward system (27). Normally, the PFC controls the dorsal striatum and the NAc in habit responses and therefore inhibits incentive salience (28). However, drug addiction disrupts the PFC leading to compromised executive functions. Increased glutamate in the PFC stimulate the habit system in the dorsal striatum which contributes to impulsivity which is associated with substance seeking (29), supporting the observed dependence syndrome reported in an extensive number of BQ users (9, 30).

The Global statistics on addiction has not formally recognized BQ use as an addictive behavior (31). However, numerous studies have used the DSM-IV criteria to measure BQD (4, 7, 11, 30, 32). A very recent study evaluated betel use disorder (BUD) using DSM-5 criteria for substance use disorders (33). In the DSM-5, substance use disorder is a combination of the DSM-IV categories of substance abuse and substance dependence into a single disorder ranging in a continuum from mild to severe (34). Changes incorporated in the DSM-5 diagnostic criteria include the presence of 2–3 symptoms from a list of 11 (the DSM-IV required only one symptom for substance abuse); an addition of drug craving and a removal of problems with the law enforcement criteria (35). Majority of current BQ neuroimaging studies have utilized Betel quid dependence scale (BQDS) to screen for BQD (36–42). BQDS was developed from DSM-IV criteria for substance dependence. It has a cut-off point of 4 and is structured into three parts; “physical and psychological urgent need,” “increasing dose,” and “maladaptive use.” BQDS has a high internal consistency (Cronbach's α = 0.921), optimal sensitivity and specificity of 0.26 and 0.977 respectively with an overall predictive accuracy up to 99.3% (8). The betel nut dependence scale (BNDS) is another measurement that has been used in neuroimaging and other related BQ studies (43, 44). The BNDS is comprised of three elements: craving and desire, withdrawal and response, and tasting habits. The scores range from 11 to 44, the higher the score the higher the level of BQD. It has a good criterion-related validity and the α coefficients of reliability lied between 0.73 and 0.89. The three elements accounted for 63.10% of total variances (45).

A number of neuroimaging modalities have been used in BQ studies including resting state functional magnetic resonance imaging (fMRI), diffusion tensor imaging (DTI), and structural MRI. Correlates of structural and functional connectivity (FC) alterations associated with BQ chewing and dependence have also been documented (36–38, 43, 46). To date, majority of published reviews have focused on neuroimaging studies of alcohol and other substance use disorders 47–51. Reviews of BQ have specifically investigated the pharmacology of intoxication and addiction (7), the association with oral cancers (52), and systemic health effects (53). There has been no systematic review of BQ neuroimaging studies to date. The purpose of this systematic review is to present the current state of knowledge in brain FC, and structural alterations associated with BQ chewing, studies' limitations and future research directions in BQ neuroimaging research.

### Objectives

We sought to identify from BQ neuroimaging findings the systems in the brain with structural, biochemical, and FC alterations related to BQ chewing.

### Research Question

What are the BQ neuroimaging findings regarding systems in the brain with structural, biochemical, and FC alterations related to BQ chewing?

## Methods

### Search Strategy

Various combination of key words was used including: betel quid, resting state, fMRI, structural MRI, betel quid dependence, and DTI which were included basing on preferred reporting items for systematic review and meta-analysis (PRISMA) criteria (54). Our systematic review protocol has been registered (CRD42018092669) in international database of prospectively registered systematic reviews in health and social care. Initial search in PubMed with the key word “betel quid” yielded” 919 articles, “betel quid dependence” yielded 27 articles, “betel quid” and “resting state” yielded 8 articles, “betel quid” AND “resting state” AND “fMRI” yielded 6 articles, “betel quid” and “structural MRI” yielded 4 articles, while “betel quid” and “DTI” yielded 1 article. Other articles were obtained from reference lists and bibliographies of the retrieved published articles.

### Data Sources, Studies Selection, and Data Extraction

Relevant articles for review of neuroimaging studies on BQ were retrieved from PubMed and EMBASE, and Google scholar databases. All unpublished (conference abstracts) and published English articles in peer reviewed journals till March, 2018 comprising one or both gender specified, regardless of study designs, were sought. Moreover, all BQ studies with fMRI and structural MRI modalities were included in our review. Exposure of interest was BQ as a mixture of areca nut, slaked lime, piper betel leaf, Acacia catechu, flavorings with or without tobacco. Any BQ study without neuroimaging modality was excluded.

The review consisted of three main stages. We first screened articles basing on the title followed by abstract and lastly we studied in detail full text articles that addressed the study aims. The latter stage was accompanied by collection of relevant information for our review. Two reviewers independently selected the articles that met the inclusion criteria. Any observed discrepancies were discussed and resolved between reviewers prior to the final selection of the articles to be included in the systematic review.

## Results

**Figure 1:**
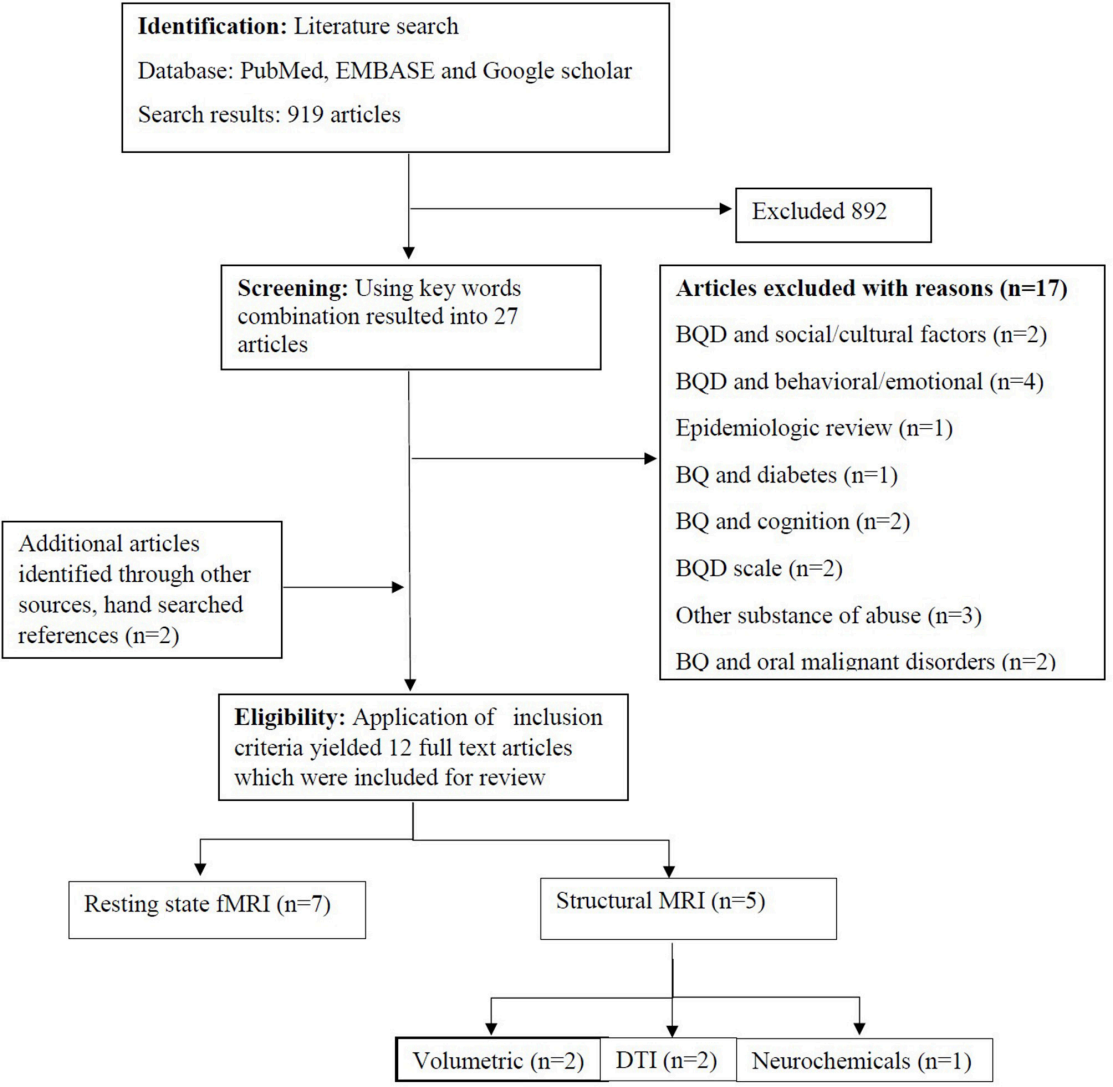
Flow diagram on included neuroimaging studies in betel quid chewers.

Of the 919 identified studies from the search results, 892 completely deviated from the established inclusion criteria, and 17 were BQ studies without neuroimaging approaches. The remaining 12 studies met the inclusion criteria (Figure 1) and were categorized according to brain functional regions including, the reward, impulsive, and cognitive systems.

### The Reward System in BQ Chewing

Neuroimaging studies have demonstrated the effects of BQ chewing and dependence in the brain reward system. Increased FC was displayed in the orbitofrontal of BQD compared to HCs (36), as well as in HCs immediately after BQ chewing (55). The BQDS scores positively correlated with the increased FC in the orbitofrontal and negatively correlated with decreased FC in the medial frontal/ACC networks (36). Similarly, some parts of the reward areas in the midbrain including, the ventral tegmental area and pons, caudate, and thalamus displayed increased FC in the BQD group compared to the controls (39). Compared with controls, gray matter (GM) volume of BQD patients was significantly reduced in the midbrain, which negatively correlated with the BQDS scores (40). A summary of BQ neuroimaging studies is presented in Table 1.

**Table 1:** Characteristics of betel quid (BQ) neuroimaging studies.

**Source**	**Setting**	**Sample/sample size**	**Sex/Age**	**Neuroimaging approach**
Weng et al. (44)	Taiwan	48 (16 BQ chewers, 15 tobacco & alcohol-users&17 HCs)	Male and female, >20 years of age	r-s fMRI
Yuan et al. (56)	Hunan province, China	54 (26 BQD & 28 HCs)	Male and female, >20 years of age	DTI
Zhu et al. (46)	Hunan province, China	54 (26 BQD & 28 HCs)	Male and female, >20 years of age	r-s fMRI
Weng et al. (43)	Taiwan	48 (16 BQ chewers, 15 tobacco & alcohol-users&17 HCs)	Male, >20 years of age	DTI
Chen et al. (40)	Hainan province China,	65 (33 BQD volunteers & 32 HCs)	Male and female, >20 years of age	sMRI
Liu et al. (39, 41, 42)	Hainan province, China	65 (33 BQD volunteers & 32 HCs)	Male and female, >20 years of age	r-s fMRI
Liu et al. (38)	Hainan province, China	65 (33 BQD volunteers & 32 HCs)	Male and female, >20 years of age	MRS
Huang et al. (55)	Hunan province, China	27 Healthy individuals	Male, 18–30 years of age	r-s fMRI
Huang et al. (36)	Hunan province, China	51 (24 BQD & 27 HCs)	Male, 18–40 years of age	r-s fMRI
Yuan et al. (37)		50 (25 BQ chewers & 25 HCs)	Male, >18 years of age	sMRI

*BQ, betel quid; HCs, healthy controls; BQD, betel quid dependence; r-s fMRI, resting state functional magnetic resonance imaging; DTI, diffusion tensor imaging; sMRI, structural magnetic resonance imaging; MRS, magnetic resonance spectroscopy*.

### The Impulsive System in BQ Chewing

A resting state fMRI study revealed a decrease in FC in the ACC of healthy males immediately after BQ chewing (55). Moreover, compared to HC, BQD individuals displayed a decreased FC in the ACC (36, 39, 46). The BQDS scores positively correlated with increased FC of right ACC to the left thalamus and left ACC to pons while the duration of BQ chewing negatively correlated with FC in the right ACC to left precuneus (39). The BQDS scores also negatively correlated with the decreased FC in medial frontal/ACC networks (36), and the right ACC (41). While investigating alterations in long and short-range FC density (FCD), a study found reduced long-range and short-range FCD in the right ACC in the BQD group compared with HCs. The short range FCD alterations in the right ACC negatively correlated with the BQDS scores (42). Studies have also documented metabolic changes that emanate from BQ chewing. For example, a magnetic resonance spectroscopy (MRS) study investigating biochemical changes in BQD chewers found reduced N-acetylaspartate/creatinine (NAA/Cr); increased choline/creatinine (Cho/Cr) and glutamate/creatinine (Glx/Cr) ratios in the bilateral ACC as well as higher Myoinositol/creatinine (MI/Cr) ratios in the left ACC of the BQD individuals compared to the control group (38). The NAA/Cr ratios in the right ACC negatively correlated with BQDS scores and BQ duration, while the NAA/Cr ratios in the left ACC negatively correlated with BQ duration. Furthermore, the NAA/Cr ratios in the right ACC positively correlated with BQDS scores (38).

Likewise, the GM in the ACC of the BQD group in a voxel-based morphometry study demonstrated significant decreased volume compared to the control group. The GM volumes of the right ACC negatively correlated with BQD duration (40). During the investigation of white matter integrity alterations, findings revealed that a smaller fractional anisotropy (FA) but larger mean diffusivity (MD) were displayed in bilateral anterior thalamic radiation (ATR) of BQD individuals compared to HCs. Both the increased MD and reduced FA correlated with severity of BQ dependence as measured by the BQDS (56).

### The Cognitive System in BQ Chewing

Neuroimaging studies have reported extensive alterations in brain functioning and structure that have been linked with cognitive impairment in BQ chewers. For instance, increased FC was observed in the cerebellum (49, 50), occipital/parietal (36, 55), occipital/temporal (55), frontoparietal, and frontotemporal networks, while decreased activity was found in the medial frontal cortex (49) and para hippocampal/hypothalamus (39). Similarly, BQD group demonstrated increased short-range FCD in the left cerebellum posterior lobe (CPL) while increased long-range FCD was observed in the left CPL and inferior parietal lobule (IPL) than the HCs. The long-range FCD alterations in the left IPL positively correlated with BQD duration. Furthermore, the BQD group displayed reduced short-range FCD in the left dlPFC compared to HCs (42). The effect of BQ was also reported by Liu et al. (41), who showed that, individuals with BQD had greater ALFF and ReHo values in the primary motor cortex area, the temporal lobe and some parts in the occipital lobe than the HCs. The BQD individuals also displayed reduced ALFF and ReHo values in the prefrontal gyrus compared to HCs (41). Apart from functional alterations, studies have also detected structural alterations in brain areas responsible for cognitive functions. Several areas in the PFC of BQ chewers including the bilateral dlPFC (37, 40), ventral medial PFC (vmPFC), and left orbitofrontal cortex (OFC) displayed reduced GM volume compared to HCs (37). The GM volumes in the dlPFC negatively correlated with BQD duration (40), and predicted the BQDS scores, history of BQ chewing and the level of daily BQ chewing (37). Reduction in GM volume was also demonstrated in the right superior temporal gyrus (STG) while increased GM volume was observed in the right hippocampal of BQD patients compared to the control group (40). Conversely, another study reported increased FC in the right hippocampus in BQ chewers compared to tobacco and alcohol users, and HCs (44). The effects of BQ chewing in the default mode network (DMN) have also been documented. For example, healthy individuals displayed reduced FC in the DMN immediately after BQ chewing (55). Additionally, compared to HCs, BQD chewers demonstrated a decrease in FC in the anterior part of the DMN comprising, the orbital mPFC/ACC, and vmPFC. The FC in the orbital mPFC/ACC in BQD individuals negatively correlated with BQDS scores (46). The BQD individuals also displayed decreased connectivity from regions in the ACC to the DMN when compared to HCs (39). The precuneus, which forms part of the DMN exhibited increased GM volume in BQD patients compared to the control group (40), which is in contrast to another study that displayed greater FC in the precuneus compared to tobacco and alcohol controls and HCs (44). Table 2 presents a summary of brain areas with altered structure, metabolism, and function in BQ chewers.

**Table 2:** Brain areas with alterations in BQ chewers.

**Source**	**Area of brain alteration**	
**FUNCTIONAL CONNECTIVITY (FC) ALTERATIONS**		
Weng et al. (44)	**↑**In bilateral precuneus and right hippocampus	**↓**Bilateral insula
Huang et al. (55)	**↑** Orbitofrontal, left frontoparietal, visual, right frontparietal, frontotemporal, occipital/parietal, occipital/temporal/cerebellum	**↓** Anterior DMN and medial front frontal/ACC
Zhu et al. (46)	NA	**↓** Anterior part of the DMN including vmPFC, (OMPFC)/(ACC).
Huang et al. (36)	**↑** Orbitofrontal, bilateral frontoparietal, frontotemporal, occipital/parietal, frontotemporal/cerebellum, and temporal/limbic networks	**↓** Parietal and medial frontal/ACC, networks
Liu et al. (39)	**↑** Long-range FCD in CPL and bilateral IPL	**↓** Long range FCD **i**n the right ACC
	**↑** Short-range FCD in the left CPL	**↓** Short range FCD in the right ACC and left dlPFC
Liu et al. (41)	**↑** ALFF and ReHo values in the primary motor cortex area, temporal lobe, and some regions of occipital lobe.	**↓** ALFF and ReHo values in the prefrontal g*y*irus along with left fusiform
Liu et al. (42)	**↑** FC from ACC to the regions of the reward network (brainstem including cortex and precuneus) and para midbrain regions such as the ventral tegmental area and pons, caudate, Hippocampal/hypothalamus.	**↓** From ACC to the DMN (medial prefrontal thalamus) and cerebellum.
**GRAY MATTER ALTERATIONS**		
Yuan et al. (37)	NA	**↓** GMV in bilateral vmPFC, bilateral dlPFC/insula, and left OFC
Chen et al. (40)	**↑** GMV in right hippocampal and right precuneus	**↓** GMV in in the midbrain, right ACC, dlPFC, and rSTG
**WHITE MATTER ALTERATIONS**		
Weng et al. (43)	**↑** Diffusion anisotropy in the ACC, midbrain, bilateral angular gyrus, rSTG, bilateral superior occipital gyrus, left middleoccipital gyrus, bilateral superior and inferior parietal lobule, and the bilateral postcentral, and precentral gyrus	NA
	**↑** Bilateral anterior cingulum	
Yuan et al. (56)	**↑** MD in ATR	**↓** FA in ATR
**BIOCHEMICAL ALTERATIONS**		
Liu et al. (38)	**↑** Cho/Cr and Glx/Cr in bilateral ACC	**↓** NAA/Cr in bilateral ACC
	**↑** mI/Cr only in the left ACC

*↑, Increased; ↓, decreased; NA, not applicable; DMN, default mode network; PFC, prefrontal cortex; OMPFC, orbirtal medial prefrontal cortex; vmPFC, ventral medial prefrontal cortex; ACC, anterior cingulate cortex; FCD, functional connectivity density; dlPFC, dorsal lateral prefrontal cortex; IPL, inferior parietal lobe; CPL, cerebellum posterior lobe; ALFF, amplitude of low frequency fluctuation; ReHo, regional homogeneity; FC, functional connectivity; Cho, choline; Cr, creatine; Glx, glutamate; mI, myo-inositol; NAA, N-acetylaspartate; GMV, gray matter volume; OFC, orbital frontal cortex; rSTG, right superior temporal gyrus; MD, mean diffusivity; ATR, anterior thalamic radiation; FA, fractional anisotropy*.

The studies included in the review were similar in that they were all BQ studies that used neuroimaging techniques to explore different alterations in the brain. However, we noted several inconsistencies among them: first, the sample sizes ranged from 27 to 65 individuals and the number of individuals in the BQD and control groups varied among studies (e.g., 16 and 33 were the minimum and maximum numbers of BQD individuals respectively). Second, majority of studies in the review reported an enhanced reward system (36, 39, 43, 44, 55) and impaired inhibitory control (36, 38–40, 42, 46, 55) in BQD chewers; (43, 44), however, are exceptions. These studies reported that BQ chewing was associated with a facilitated inhibitory control. The observed disparity may have been influenced by the variation in scales used to identify individuals with BQD (that is, BNDS in the latter two studies vs. BQDS in the former studies) leading to the inclusion of subjects with different BQ dependence levels. Moreover, examining only 16 individuals in the BQ chewers group may have made it difficult to identify clearly brain areas with alterations. Likewise, the inclusion of controls who used tobacco and alcohol concomitantly may have influenced the results, possibly due to the different neurobiological effects these substances tend to impose on the brain.

### Risk of Bias

We assessed for risk of bias in the included primary studies by considering the sample size, gender, inclusion criteria for individuals with BQD and controls, BQD screening tools, and other potential sources of bias to determine how these may have affected the study results. To prevent publication bias, we considered both published and unpublished studies in our search strategy. Any observed discrepancies were discussed and resolved between reviewers prior to the final selection of the articles to be included in the systematic review.

## Discussion

In this systematic review, we were able to identify 12 neuroimaging studies which have highlighted the current state of knowledge about important brain systems with structural, metabolic, and functional alterations associated with BQ chewing.

### The Reward System in BQ Chewing

Humans tend to consume alcohol or self-administer drugs of abuse because they experience rewarding effects from these substances (22). The reward areas in the brain include the basal ganglia, the limbic system, and parts of the PFC (57). The review found increased FC in the orbitofrontal cortex which forms part of the PFC. The orbitofrontal network is known for its function in regulating emotions, monitoring reward and evaluation of punishers (58) whose disruption by addictive drugs is related to maladaptive and impulsive decision making (59). It has also been suggested that the OFC together with the dlPFC are involved in reward processing (60), explaining the observed decision making and goal driven behavior abnormalities in BQ chewers (41). Additionally, decreased FC in vmPFC and orbital mPFC in BQD supports the addiction model (26, 61), where they have been reported to play a role in salience attribution and goal directed behaviors (62). Increased connectivity (39) and diffusion anisotropy (43) was observed in midbrain regions of BQ users. DA neurons in the midbrain play an important role in attention, motivation, motor control, regulation of emotion, maintenance of working memory, and reinforcement (63). The DA cells in the VTA projecting into the NAc (the mesolimbic DA pathway), those in the substantia niagra (SN) projecting into dorsal striatum (mesostriatal) as well as those in the VTA projecting in to the frontal cortex (mesocortical) play a very important role in drug reward and addiction (22). During addiction, there is a higher expectation value of the drug in the reward, memory, and motivation circuits which overcomes the control circuit leading to consumption of drugs (64). Task-based fMRI studies have reported midbrain activation during anticipation of pleasant tastes (65), monetary gains (66), and exposure to visual stimuli that elicited feelings of intense romantic love (67). The dorsal striatum in the midbrain plays a fundamental role in acquisition and expression of action–outcome associations conditioning (68) including the development of habitual compulsive drug dependence. Brain neuroimaging studies showed that increased levels of DA in the dorsal striatum (caudate and putamen) were triggered by alcohol-associated cues, an effect that correlated with self-reports of craving for alcohol (69). Significant increased FC was displayed in the cerebellum of BQD individuals. The cerebellum also plays part in reward monitoring and as an intermediate between motor and reward, as well as motivation and cognitive control systems, which are all relevant etiologic factors in drug addiction (70). Studies have also reported extremely increased glucose metabolism in the cerebellum of addicts when they performed reward expectation tasks (71). Activation of the cerebellum has been directly related to the intensity of cue-elicited craving (72).

### The Impulsive System in BQ Chewing

The PFC, OFC and ACC are known for their contribution in executive functions and impulse inhibition (73). Alterations in the PFC as a result of addiction leads to compulsive drug taking and detrimental behaviors which are associated with addiction and loss of free will (28). Studies have reported about the reduction of striatal dopamine 2 receptors (D2R) in addicted individuals after extended period of detoxification (74). The reduced striatal D2R reductions have been linked with decreased metabolism in the OFC, dlPFC, and ACC (75), whose role in salience attribution, decision making, and emotional regulation/inhibitory control form a basis to the enhanced motivational value of drugs in their behavior and loss of inhibition to drug use (76). Evidence also shows that, disrupted OFC and ACC are associated with impulsivity behaviors (77). For instance, individuals who abused methamphetamine displayed reduced striatal D2R which was linked to impulsivity (78). The limbic network is implicated in reward processing, addiction, and goal-directed behavior (79), supporting the increased FC reported from the BQ neuroimaging studies. Activation of the limbic networks and the loss of inhibition in the frontal cortical areas may lead to impulsivity, which is a major characteristic of addiction (73). The ACC forms part of the DMN, and it was observed to have a reduction in connectivity (36, 39, 42, 46, 55), GM volume (40), and NAA/Cr (38). In addiction theories, ACC is thought to be involved in inhibitory control of reward-related behavior (61). As a result, addicted individuals are unable to control short term and immediate gratification of habitual drug use regardless of existing or anticipated consequences (59). Reduced activation in the ACC of opiate-dependent individuals has been linked with deficits in response inhibition and impulse control (80). Reduction in NAA concentration was reported in the dorsal ACC (81) and frontal gray matter (82) of opiate dependent individuals. This reduction has often been interpreted as a representation of neuronal damage and/or loss (83, 84). Decreased NAA levels in the ACC of BQD individuals supports preclinical work, signifying that Arecoline leads to neurotoxicity with greater oxidative stress and suppressed antioxidant protective system (85). Increased levels of Cho/Cr, Glx/Cr, and ml/Cr in the ACC of BQD individuals was reported by Liu et al. (38). The increased Cho/Cr levels portrays a high intracellular Cho in ACC neurons which may be the pathological phenomenon or compensatory response to BDQ. Studies have shown that ml is an osmoregulator (86), and elevated levels of mI were reported in the ACC of alcoholic patients, which indicates a temporarily increased glial activation or, a state of drug-induced osmotic stress, and an attempt to regulate cell volume (87). Meanwhile, Glx increase is considered a potential illustration of abnormalities in decision making and reward-based learning, or a modulator of dopaminergic neurotransmission (81). Reduced FA in the ATR of BQD individuals was reported by Yuan et al. (56). The ATR (links the anterior and medial thalamic nuclei with the PFC) is known for its role in modulating the basic impulses and flexibility in attaining planned goals, whose disruption has been linked with lack of inhibition from the PFC (88). It is also involved in controlling basic impulses and working toward defined goals (88–91). Extensive decreased white matter integrity has been reported in the ATR of pathological gamblers (92) and internet gaming disorder subjects (93). The observed alterations suggest a significant role of the ATR in the neurobiology of BQD (56). Furthermore, increased MD within the ATR of BQ dependent individuals (56) is suggested to be linked with reduced myelination or neuronal loss (94), explaining a lack of inhibition from prefrontal cortex to the subcortical areas (89). This mechanism has been mentioned in substance and behavioral addiction (88–91, 95).

### The Cognitive System in BQ Chewing

Several areas of the brain are involved in cognitive processing including the PFC [orbital frontal cortex (OFC), dorsal lateral PFC (dlPFC), ventral medial PFC (vmPFC)], limbic regions, cerebellum, parietal, occipital, and temporal lobes. The PFC participates in various cognitive processes, such as working memory, attention, decision making and delay discounting, all of which are impaired in addicts (28). BQ neuroimaging studies have reported increased connectivity in the frontoparietal, frontotemporal, occipital/parietal, occipital/temporal/cerebellum (36, 49). Habitual users of BQ reported experiencing heightened alertness, euphoria, relaxation, arousal, improved motor responses, and a sense of wellbeing (5), suggesting that the increased connectivity may enhance cognitive abilities (55) and thus perpetuate addiction behavior. BQ chewing also displayed reductions in FC (39, 41, 42, 46) and GM volume (39, 52) in the PFC including the dlPFC, mPFC, vmPFC, and orbital mPFC. In addiction studies, the PFC plays a crucial role in self-control activities, salience attribution, preservation of motivational arousal and self-awareness (28). Addiction studies have extensively cited the dlPFC which interacts with numerous structures in the cortex. For instance, together with the dACC, the dlPFC exerts top-down control and meta-cognitive functions (28). The dlPFC is also involved in working memory processing (96, 97), decision making and cognitive control (98, 99), executive functions (100), as well as conflict resolution (101). Therefore, the reported alterations in the dlPFC may have contributed to impaired decision making, cognitive control and memory processing leading to habitual and compulsive BQ chewing. Additionally, BQ studies reported decreased GM volume (37) and increased FC (44) in the insula in BQ chewers. The insula plays a critical role in generating conscious, interoceptive signals into what one may subjectively experience as a feeling of desire, anticipation, or urge (88, 102). Decreased connectivity of the interoceptive insula in BQ chewers may make them misadjust the reward value of the substance to optimize their choices to satisfy their internal and external need (103, 104). Neuroimaging studies have shown that addicted individuals had lower GM volume and reduced activity of insula (105).

The PFC is also linked with other cortical and subcortical brain regions and networks, including the DMN and the dorsal attention networks which are involved in executive control processes, such as attention and inhibition (106). Functional MRI studies have established that reduction in neural connectivity of the PFC is associated with maladaptive decision making (107) and cognitive control (108). The DMN which is known to be active when the brain is “at rest,” has been implicated in mind-wandering (109) and social understanding of others including, emotional perception, empathy, and morality (110). The DMN deactivates when individuals focus on the external environment or perform goal-directed tasks (111). The effects of BQ chewing in suppressing DMN connectivity have been associated with a reduction in mind-wandering, enhanced focused attention, lessened depression, improved social cognition (55), self-awareness and insight into illness (112). Neuro dysfunction in the DMN of addicts tends to compromise insight, disease awareness, and need for treatment (112), supporting what is demonstrated in BQD individuals.

The frontoparietal network is known for its involvement in cognitive control, and development of reasoning ability (113). Along with the frontotemporal, visual, and occipitoparietal regions are involved in visual spatial judgment (114) while the frontoparietal is involved in spoken language comprehension (115). Similarly, increased long range FCD in IPL was thought to impair distant information processing in BQD individuals (42). The IPL is linked with verbal fluency, working memory, complex sequential motor behavior, and skill learning (116). Verbal fluency deficits have been observed in cocaine-dependent individuals (117).

Additionally, increased connectivity between the cerebellum and other brain areas (36, 39, 42, 55) supports its role in decision making (118), and acts as a link between motivation and cognitive control systems, thus playing an important role in drug addiction (70). Reduced GM volume was found in the superior temporal gyrus (STG) (40) which is an important structure in social cognition (119) and emotion (120). There is evidence that neuronal networks connecting the mPFC with temporal areas are greatly destroyed in BQD patients, which is consistent with a previous fMRI study on cocaine users (121).

Studies have reported increased GM volume (40) and FC (44) in the precuneus and hippocampus in BQ chewers. Precuneus has been associated with identification of visual and appetite cues (122, 123), which conforms with BQ chewing, while the hippocampus is involved in processing memory and emotions (124). Studies have found that compared to non-dependent and non-chewers, BQ dependent chewers displayed impaired spatial short term memory (125). BQ chewing has also been associated with antidepressant properties due to its influence in increasing the levels of serotonin and noradrenaline (126), proposing the role of hippocampus in emotional regulation, predominantly depression. The thalamus has been increasingly implicated in addiction due to its integrative function in regulating arousal and modulating attention. An effect related to craving was observed when dopamine neurotransmission in the thalamus was increased due to intravenous administration of Methyphenidate in cocaine users as compared to controls (127). Similarly, Results from both PET and fMRI results have demonstrated that compared to non-rewards, both primary and secondary rewards can increase thalamic activation (128).

Findings from neuroimaging studies have also demonstrated alterations in white matter integrity (43) and FC in the visual cortex of BQD individuals (36, 41, 55). The visual cortex is known for its role in cognitive processing including visual perception, working memory (129) as well as influencing motivation and alertness (130). Despite its documented roles in the brain, the visual cortex has received less attention in addiction neuroimaging studies. The reported alterations in the visual cortex support the heightened alertness experienced by BQ habitual users (8). These changes are believed to originate from both acute (55) and chronic (36) effects of BQ exposure. Similar studies have found increased FC in the visual cortex (131) and specifically in the primary visual cortex during acute alcohol administration (132). Likewise, compared to neutral cues, exposure to visual drug cues elicited greater activation in the visual cortex of substance abusers (133).

### Limitations

There are however several limitations to note from these studies. First, the differences in subjects among studies (age, gender, variations in BQ preparation, dependence level, and the duration of BQ exposure) could have potentially influence the results. Second, inclusion criteria of individuals with BQD, where different screening tools (BQDS vs. BNDS) were utilized to identify individuals with BQD could have biased our results. Third, all reviewed BQ neuroimaging studies were observational, which limits inferences on causality. Future longitudinal neuroimaging studies may lead to a better understanding of mechanisms underlying BQ use and thus describe a cause and effect relationship among brain alterations and BQ use. Fourth, four (4) studies only included men as study participants which may have limited the generalizability of study findings to females. Fifth, very small sample sizes were used with a range from 27 to 65 which could have potentially led to biased results. Studies with larger sample sizes with inclusion of both males and females may improve generalizability and prevent unbiased results. Lastly the observed brain alterations cannot be deeply attributed to BQ chewing only but considerations should be taken on the possibility of the potential influence of alcohol and tobacco consumption in the results.

## Conclusion

The review has highlighted the current state of knowledge regarding important systems in the brain that are commonly affected by BQ chewing and BQD. Generally, the aforementioned brain alterations have been involved in one way or another to enhance the reward system, decline inhibitory control and executive functions (including emotions, cognition, and affective decision making) in BQ chewers and BQ dependent individuals. The exhibited brain alterations are associated with BQD severity and duration of BQ use. These alterations have all been implicated to potentially play a role in drug addiction. Therefore, further neuroimaging research involving BQ dependent and abstinent individuals might help explain the neuro-mechanisms of BQ use and hence extend what these studies found.

## Author Contributions

AS and XH searched, selected, and reviewed all articles, and wrote the first draft of the manuscript. AS, XH, ZL, WP, HL, and ZX revised and approved the final version of this manuscript.

### Conflict of Interest Statement

The authors declare that the research was conducted in the absence of any commercial or financial relationships that could be construed as a potential conflict of interest.
